# Building and rebuilding complex tissues: strategic visions from a research-led workshop

**DOI:** 10.1242/dev.205106

**Published:** 2025-08-07

**Authors:** N. Sumru Bayin, Benjamin Steventon, Mekayla Storer

**Affiliations:** 1 Gurdon Institute, University of Cambridge, Cambridge CB2 1QN, UK; 2 Department of Physiology, Development and Neuroscience, University of Cambridge, Cambridge CB2 3EG, UK; 3 Department of Genetics, University of Cambridge, Cambridge CB2 3EH, UK; 4 Cambridge Stem Cell Institute, University of Cambridge, Cambridge CB2 0AW, UK

**Keywords:** Regeneration, Community building, Non-model organisms, Comparative analysis

## Abstract

How complex tissues develop and regenerate post-injury is one of the most fascinating and important processes in biology. Recent technical advances that enable the generation of a quantitative understanding of these processes have the potential to transform the field. However, to achieve this, concerted and long-term studies are required at the community level to compare regeneration and wound healing in both regenerative and non-regenerative contexts and organisms. To stimulate such collaboration, we recently organised a focused workshop around this topic to identify key outstanding questions that focus on cross-comparisons of regenerative competence, and the cellular, molecular and physical drivers of regeneration. Importantly, the discussions also highlighted significant logistical and financial challenges in initiating and sustaining large-scale, long-term interdisciplinary studies, particularly those involving non-model organisms.

## Introduction

One of the most captivating questions in the field of developmental biology is the ability of complex tissues to regenerate and re-establish their function upon injury. Striking examples of this capacity exist within multiple species, yet it is a capacity that is broadly limited in our own biology (Losner et al., 2021). Therefore, unlocking the secrets of what is required for complex tissue regeneration requires a concerted, interdisciplinary effort across the community to compare regenerative and non-regenerative systems (Seifert et al., 2023). This is no easy task, given the full range of biological events that must be coordinated during organ regeneration (Poss and Tanaka, 2024). The rapid development of systems-level technical approaches to canvass cellular function with single-cell resolution provides great promise for the field (Griffiths et al., 2018). Similarly, the increasing interest in understanding the role of the cellular niche that signals to the regenerating cells and the acellular niche in terms of matrix composition and mechanical properties is opening new questions in the field (Mao and Wickström, 2024). Finally, the clear role of metabolic switches in response to injury makes regeneration one of the best-studied examples of metabolic control in developmental processes (Eming et al., 2017). However, most regenerative biology research is done in silos, focused on individual regenerative phenomena and lacking systematic comparative analysis across systems and species. Given the full breadth of these challenges, we reasoned that a community-driven approach is required to make significant advances in the field. Therefore, to facilitate collaboration, we organised a two-day event. The first day featured an international symposium with 12 invited speakers, while the second day was dedicated to interactive workshop sessions focused on addressing major open questions and identifying priority research areas. In this Perspective, we describe the workshop format and summarise the key discussion outcomes and challenges identified.

## Complex Tissue Regeneration Grand Challenge

To eliminate departmental boundaries and connect interdisciplinary researchers interested in similar questions, the School of Biological Sciences at the University of Cambridge has identified six research themes with the aim of identifying grand challenge ideas for each of these themes. The ‘Complex Tissue Regeneration Grand Challenge’ is one of these endeavours to unite regeneration researchers across the university, together with the long-term goal of accumulating critical mass and strategic funding to be at the forefront of the developments in this area. As a part of this Grand Challenge, in July 2024, we hosted UK and world leaders at Robinson College for our ‘Building and Rebuilding Complex Tissues Meeting and Workshop’.

The primary goal of this workshop was to identify open questions in the regenerative biology field and facilitate new collaborations. While the symposium was open registration, the workshop was restricted to selected individuals to facilitate discussion amongst interested parties. Participants included conference invited speakers and regeneration researchers across the University of Cambridge. To maximise use of our time, prior to the workshop, all participants were given a worksheet requiring them to identify and prioritise open questions and areas of collaborative needs, as well as challenges being faced in their immediate fields of research.

## Workshop structure

### Science sparks: speed networking to fuel interdisciplinary conversations

Encouraging interdisciplinary collaboration can be both rewarding and challenging, especially when researchers come from vastly different academic backgrounds. For meaningful ideas to be shared, participants must feel at ease in a safe and welcoming environment, and, importantly, with each other. Here, we instigated scientific speed networking at the beginning of the workshop, providing a structured yet informal setting for rapid, one-on-one conversations. Participants were seated on either side of long tables, with one side remaining stationary while the other rotated after each timed interaction. With just a few minutes per interaction, participants were able to share their work, identify common interests, and begin to build connections that might not have emerged through traditional networking formats. We found this approach was highly successful in promoting intellectual exchange, but, more importantly, played a key social role in breaking the ice, reducing initial awkwardness, and helping participants feel more comfortable engaging across disciplinary boundaries.

### Using flash talks as a gateway to research visibility

As part of the workshop, each participant delivered a 2-minute flash talk using a single slide to briefly introduce their research. These concise presentations served as a quick yet effective way for researchers to share the core focus of their work, the techniques they employ, and the open questions they are exploring. By encouraging clarity and accessibility, the flash talks helped create a shared understanding among participants from diverse backgrounds. To maintain energy and engagement throughout the day, the talks were strategically dispersed between sessions, allowing for continued exposure to new ideas and facilitating meaningful conversations and collaborations during breaks.

### Exploring key themes through focused group discussions

A significant portion of the workshop was devoted to discussion sessions structured around four key research areas and challenges in regenerative biology, which were identified through a pre-workshop survey. The discussions took place in rotating small groups, where participants shared their interests, expertise and current challenges to identify key hypotheses and potential opportunities for collaboration.

#### Microenvironment and regenerative niches

Cells do not function in isolation. Complex tissue development and regeneration involve many different lineages within the parenchymal and stromal components of organs and tissues. In addition, an understanding of various inter-organ modalities, such as nutrient and oxygen availability, is needed to resolve how tissues are built and rebuilt. Therefore, there was a broad consensus on the need for holistic approaches that take the microenvironment into account. Concurrently, the discussions highlighted the need for improved tools to integrate multimodal datasets in a manner that preserves spatial information.

#### Size sensing and morphology

An important aspect of development and regeneration is to ensure that the correct morphology and size are achieved at the end of these processes. However, the molecular and physical mechanisms driving size sensing and morphology are poorly understood in complex tissues. Gaining such understanding requires a multidisciplinary approach between developmental and regenerative biologists and biophysicists. Importantly, we discussed whether achieving correct morphology is crucial for regeneration or whether we should shift our perspective towards prioritising the recovery of organ/tissue function.

#### Cross-species comparisons of developmental mechanisms and regenerative capacity

One of the strengths of our field is the plethora of model and non-model organisms that we use to study how tissues are built and rebuilt. While the field appreciates the need for diverse model and non-model organisms, as well as comparative analyses to understand why regeneration succeeds in some systems but not others, conducting these studies in an unbiased manner and extrapolating these findings remains a significant challenge. Addressing this challenge requires close collaboration with computational biologists and data scientists. For example, for reliable comparisons, different developmental/regenerative states across species and organs are needed. Therefore, we discussed how we might leverage AI and machine-learning tools to compare morphological characteristics across different species or generate useful staging tables for such comparisons. Such approaches also require community engagement and open science practices.

#### Changes in regenerative capacity across time or with ageing

An overarching theme in most of our systems was the decline in regenerative potential over time. This is a complex, multifaceted problem involving cell-intrinsic mechanisms that limit the regenerative potential of cells, as well as changes in microenvironment and organismal physiology, and, likely, further complicated by hallmarks of ageing. We discussed strategies for systemically studying these temporal changes and, similar to the previous question, how to ensure that comparisons across time – and across different biological scales – can be made reliably.

### Challenges identified and outlook for the field

One of the main outcomes of the workshop was the recognition that advancing our understanding of complex tissue regeneration requires long-term strategic funding, and often at the consortium level. Additionally, enabling meaningful comparisons across biological scales depends on targeted training programmes that support skill transfer. The challenges of working with large datasets also highlight the need for unified data collection and a commitment to open science practices. Importantly, advances require researchers to take bold steps into the use of non-model organisms for experimental studies of regeneration. While this approach is often fraught with inherent risk, it is essential for overcoming the limitations of traditional model systems and addressing the logistical and ethical challenges of whole-animal research. In parallel, there is a need for global research projects that engage with local communities in ethical, sustainable ways that enable the discovery of new regeneration paradigms. We agreed that large-scale cross comparisons are required to uncover the underlying principles that govern regenerative capacity. Research in this area would be very timely, as it leverages the use of advanced technologies at the systems level to uncover core principles of regeneration and development.
